# Association of Interferon Gamma +874T/A Polymorphism and Leukemia Risk: A Meta-Analysis

**DOI:** 10.1097/MD.0000000000003129

**Published:** 2016-03-25

**Authors:** Zhitong Wu, Yifan Sun, Shengbo Zhu, Shifu Tang, Chunming Liu, Wenzhou Qin

**Affiliations:** From the Department of Clinical Laboratory (ZW, WQ), Guigang City People's Hospital, Guigang; and Department of Clinical Laboratory (YS, SZ, ST, CL), Liuzhou Hospital of Traditional Chinese Medicine, Liuzhou, Guangxi, China.

## Abstract

Interferon gamma (IFN-γ) has antitumor and antiproliferative effects, and previous studies indicated *IFN-γ* +874T/A (rs2430561) polymorphism were related to the risk of many types of cancer. However, the association between *IFN-γ* +874T/A polymorphism and leukemia risk remained controversial.

We performed a comprehensive meta-analysis based on the Preferred Reporting Items for Systematic Reviews and Meta-analyses statement (PRISMA). Electronic database of Embase, Pubmed, and the Cochrane Library were searched for eligible articles published up to December 13, 2015. The association between genetic polymorphisms and leukemia risk was measured by odds ratios (ORs) and its corresponding 95% confidence intervals (CIs).

A total of 8 studies amounting to 420 patients and 767 control subjects were retrieved for this study. Although associations between *IFN-γ* +874T/A polymorphism and overall leukemia risks were lacking, decreased chronic lymphocytic leukemia (CLL) risk was detected in the allelic model (T vs A, OR=0.660, 95%CI = 0.483–0.902, *P* = 0.009, *I*^2^ = 0.0% and *P* = 0.863 for heterogeneity), the codominant model (TT vs AA, OR = 0.472, 95%CI = 0.247–0.902, *P* = 0.023, *I*^2^ = 0.0% and *P* = 0.994 for heterogeneity), and dominant model (TT + TA vs AA, OR = 0.457, 95%CI = 0.285–0.734, *P* = 0.001, *I*^2^ = 40.3% and *P* = 0.195 for heterogeneity) by using fixed-effect model separately. On the contrary, results indicated T carries have an increased chronic myelogenous leukemia (CML) risk in dominant model (TT + TA vs AA, OR = 1.783, 95%CI = 1.236–2.573, *P* = 0.002, *I*^2^ = 19.0% and *P* = 0.295 for heterogeneity).

This study suggests *IFN-γ* +874T/A polymorphism are related to CML and CLL risk. In addition, our work also points out IFN-γ +874T/A polymorphism may play dual contrasting role in leukemia risk.

## INTRODUCTION

Leukemia is one of the most common malignancy in human, with an estimated 368,800 new cases and 303,200 deaths occurred in 2012 worldwide.^1^ Moreover, leukemia is the leading cause of cancer death in those aged younger than 40 years among males and in children and adolescents (aged younger than 20 years) among females.^2^ It is generally considered that both environmental and genetic risk factors contribute to leukemic progress; however, the biological mechanisms of leukemia are complex and have not been fully clarified. Cytokines and their genes play a fundamental role in the immunopathogenesis of malignant, infectious, and autoimmune disease.^3–5^ It is, therefore, important to identify these genes variants contributing to leukemia pathogenesis.

Interferon gamma (IFN-γ) is a T-helper 1 (Th1) proinflammatory cytokine that plays a pivotal role in antiviral activities and has antitumor and antiproliferative effects.^6^ The production of IFN-γ is under genetic control. The SNP (+874T/A, rs2430561) of *IFN-γ* gene, a T-to-A transition at +874 loci in the 1st intron of *IFN-γ* gene, is complete linkage disequilibrium with the12 CA repeat microsatellite and correlated with IFN-γ production.^7^ Moreover, the transcription factor nuclear factor kappa-B (NF-κB) binds preferentially to the +874T allele, which is known to upregulate the expression of IFN-γ.^8^ The +874T/A polymorphism generates 3 genotypes (TT, TA, and AA), and TT genotype produces a high level of IFN-γ, helps the host's defense against viral infection. Conversely, the genotypes AA and AT cause relative low IFN-γ production, which may increase the risk of viral infection.^7,8^ It appears that the +874T/A polymorphism can influence IFN-γ expression, and in this way, affects immune response.

The relationship between *IFN-γ* genes and leukemia was previously studied,^9^ in addition, a number of studies have attempted to assess associations between *IFN-γ* +874T/A polymorphism and leukemia risk.^10–16^ However, a unified conclusion is still unreached, furthermore, most of these studies had insufficient sample sizes of studied populations, which led to lower statistical power. Therefore, we conducted a meta-analysis to evaluate broadly the available evidence on the association between *IFN*-γ +874 T/A polymorphism and leukemia risk.

## MATERIALS AND METHODS

This meta-analysis was conducted according to Preferred Reporting Items for Systematic Reviews and Meta-analyses (PRISMA) statement (S1_Table), including the search strategy, selection criteria, data extraction, and data analysis.^17^

### Identification of Eligible Studies

Two investigators (YS and ZW) searched independently for all publications in Embase, PubMed, and Cochrane Library up to December 14, 2015, by using the following search terms: “interferon gamma,” “IFN-γ,” “rs2430561,” “polymorphism,” and “leukemia.” Articles in reference lists were also hand-searched. Only human studies were searched and there was no restriction on the language.

### Inclusion and Exclusion Criteria

The 2 investigators choose the suitable studies according to the following criteria:

1. Case–control or cohort design studies evaluated the associations between IFN-γ+874T/A polymorphism and leukemia risk, and the controls should be healthy populations.
2. Studies offering the ability to extract data for calculating the odds ratio (OR), 95% confidence intervals (CIs), and Hardy–Weinberg equilibrium (HWE).

The exclusion criteria were:

1. Review articles, letters, case reports, editorials, and conference abstracts.
2. Family based studies.
3. The articles did not include genotype frequencies, and this information was not available from the authors contacted.
4. Duplicate data.

### Data Extraction

The based characters and data included the first author's name, publication date, country, ethnicity, diagnosis criteria for leukemia, source of control, total sample size, genotyping method, and the genotype frequencies of cases and controls were extracted independently by the 2 investigators (YS and ZW) from the included studies. To determine the accuracy of the extracted information, the data extracted by the 2 investigators should be all the same, and if there was a dispute they should check their data again. If the 2 investigators could not reach an agreement, the dispute was submitted to a 3rd reviewer (CL) to decide. In addition, if the genotype frequencies of cases and controls could not be extracted directly from the article, the 2 investigators should try to contact the authors for help, or obtain the data from previous literatures. HWE was calculated according to the genotype frequencies of controls. The quality of the studies included in this meta-analysis was assessed according to HWE, and we considered a high quality study should be fall in HWE.

### Statistical Analysis

The association between genetic polymorphisms and leukemia risk was measured by ORs and its corresponding 95% CIs. The allelic model (T vs A), codominant model (TT vs AA, TA vs AA), dominant model (TT + TA vs AA), and recessive model (TT vs TA + AA) were examined.

Heterogeneity was assessed by a Chi-squared *Q*-test and *I*^2^ statistics.^18,19^ The fixed-effects model (the Mantel–Haenszel method) is used when the effects are assumed to be homogenous (*P*_Q_ ≥ 0.1 or *I*^2^ < 50%). Otherwise, the random effects model (the DerSimonian and Laird method) is used when they are heterogeneous (*P*_Q_ < 0.1 or *I*^2^ ≥ 50%). Subgroup analysis was carried out stratified by leukemia type, ethnicity (Caucasian and Asian), and HWE (fall in HWE). Begg's funnel plot and Egger's test were used to investigate the publication bias in the meta-analysis; *P* < 0.05 indicated that the result was statistically significant. Sensitivity analysis was performed by omitting 1 study at a time.

All the tests in this meta-analysis were conducted with RevMan 5.2.7 (Cochrane Collaboration) and STATA software (version 12.0; Stata Corporation, College Station, TX).

An ethical approval was not necessary needed as this is a meta-analysis based on previous studies.

## RESULTS

### Study Characteristics

**FIGURE 1:**
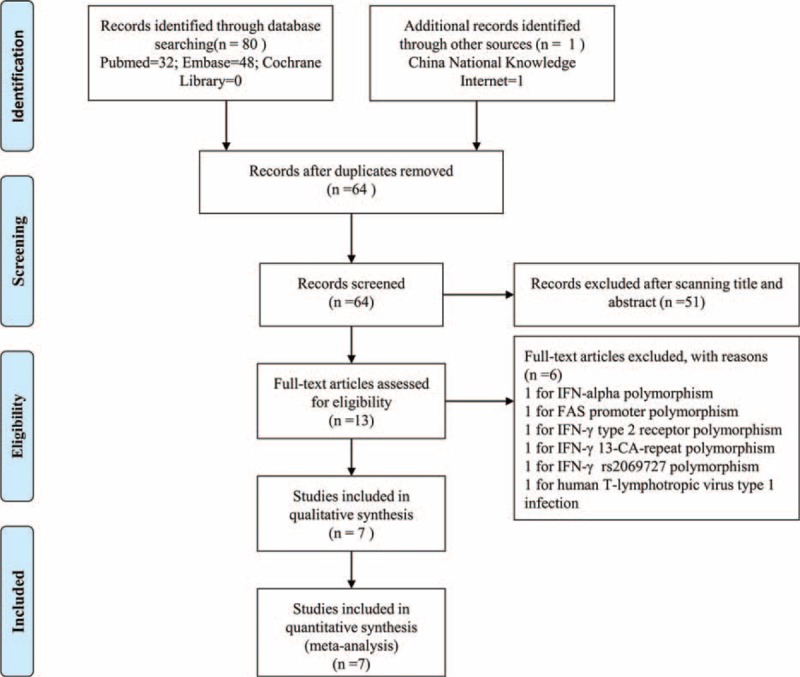
Flow diagram of included studies for this meta-analysis.

The literature search and study selection procedures are shown in Figure 1. Leukemia types, genotype numbers, and HWE examination results are shown in Table 1. According to the inclusion and exclusion criteria, 7 articles were included in the meta-analysis. The article performed by Baştürk et al^11^ was separated as 2 studies for they evaluated 2 different diseases, therefore, a total of 8 studies amounting to 420 patients and 767 control subjects were retrieved for this study. Two studies populations come from Asian^10,12^ and 6 studies were Caucasian.^11,13–16^ Two studies were failed to fall in HWE.^10,14^

**TABLE 1:**
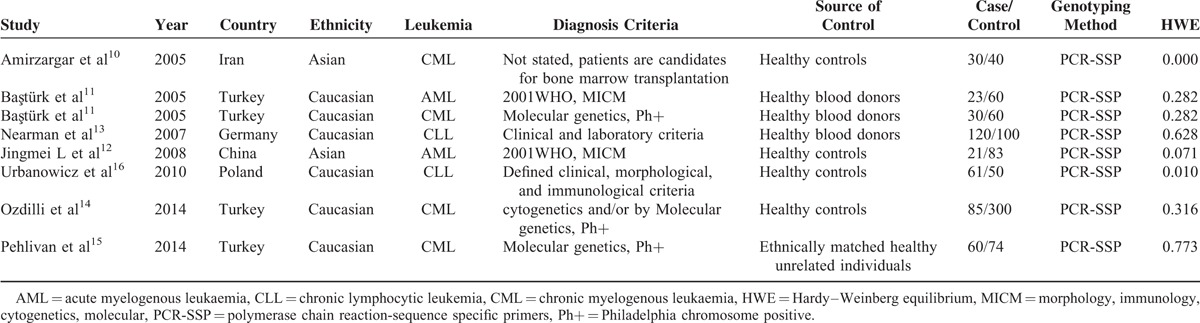
Characteristics of Studies Included in the Meta-Analysis

### Meta-Analysis for the Association Between the *IFN-γ* +874A/T Polymorphism and Leukemia

The meta-analysis results for all the studies are shown in Table 2. When all the eligible studies were pooled into the meta-analysis of *IFN-γ* +874A/T polymorphism, there was no significant association between *IFN-γ* +874A/T polymorphism and leukemia risk under all comparison models.

**TABLE 2:**
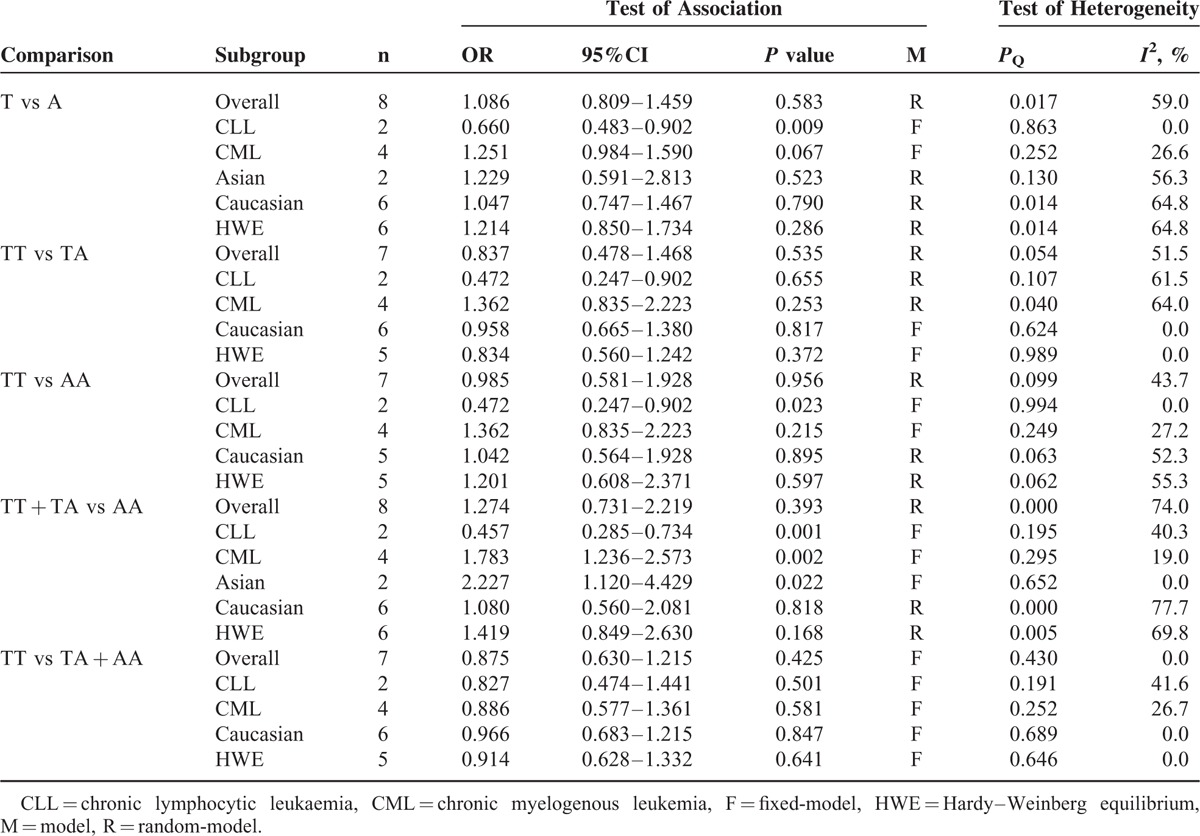
Meta-Analysis of Interferon Gamma +874T/A Polymorphisms and Leukemia Risk

**FIGURE 2:**
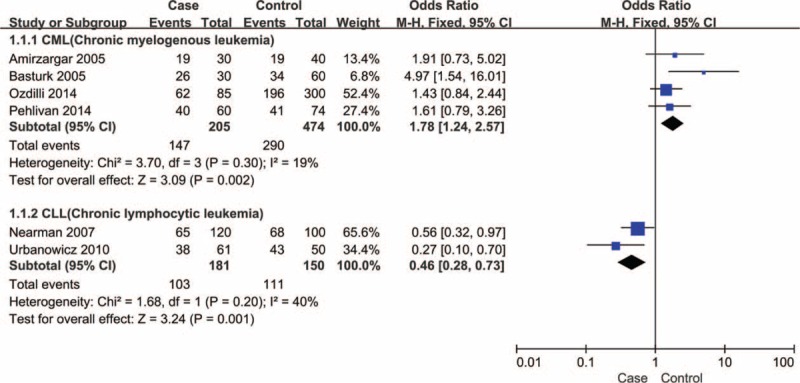
Forest plot for the association between IFN-γ (+874T/A) polymorphism and leukemia risk stratified by leukemia type in a dominant model (TT + TA vs AA) using a fixed-effects mode. The squares and horizontal lines correspond to the study-specific OR and 95%CI. The diamond represents the summary OR and 95%CI. CI = confidence interval, IFN-γ = interferon gamma, OR = odds ratio.

However, significant heterogeneity between studies was detected in comparison model expect recessive model. Therefore, we further performed subgroup analysis (Table 2). In the subgroup analysis by leukemia type, significantly elevated chronic myelogenous leukemia (CML) risk was observed in dominant model (TT + TA vs AA, OR = 1.783, 95%CI = 1.236–2.573, *P* = 0.002, *I*^2^ = 19.0% and *P* = 0.295 for heterogeneity, Figure 2) by using fixed-effect model. However, significantly decreased risk was found among chronic lymphocytic leukemia (CLL) in the allelic model (T vs A, OR = 0.660, 95%CI = 0.483–0.902, *P* = 0.009, *I*^2^ = 0.0% and *P* = 0.863 for heterogeneity), the codominant model (TT vs AA, OR = 0.472, 95%CI = 0.247–0.902, *P* = 0.023, *I*^2^ = 0.0% and *P* = 0.994 for heterogeneity), and dominant model (TT + TA vs AA, OR = 0.457, 95%CI = 0.285–0.734, *P* = 0.001, *I*^2^ = 40.3% and *P* = 0.195 for heterogeneity, Figure 2) by using fixed-effect model separately. In the subgroup analysis by ethnicities, no significant associations were found in Caucasian group under all comparison models. However, significant associations were found in the dominant model (TT + TA vs AA, OR = 2.227, 95%CI = 1.120–4.429, *P* = 0.022, *I*^2^ = 0.0% and *P* = 0.652 for heterogeneity) among Asian group by using fixed-effect model.

### Evaluation of Heterogeneity and Publication Bias

Significant heterogeneity was discovered in this study expect the recessive model in the overall population. Then we carried out subgroup analysis according to leukemia type, and the results showed there was no obvious heterogeneity in the subgroup analyses of CLL and CML, expect TT vs TA analysis (*I*^2^ > 50%, Table 2), suggesting that leukemia type was the major source of heterogeneity in our meta-analysis.

**FIGURE 3:**
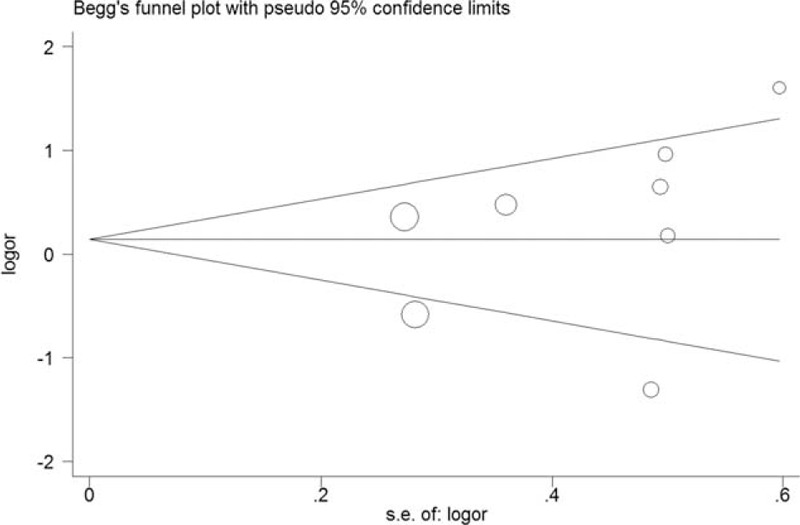
Begg funnel plot for contrast in a dominant model (TT + TA vs AA). Each point represents a separate study for the indicated association. Size graph symbol by weights. Log[OR] natural logarithm of odds ratio (OR). Horizontal line mean effect size.

Figure 3 showed the Begg's funnel plot in allelic model. No significant publication bias was detected using Begg funnel plot in the overall population in all comparison models. The statistical results of Egger's test also proved the evidence of funnel plot symmetry.

### Sensitivity Analysis

Sensitivity analysis was performed by omitting 1 study at a time, and the estimate of overall effect did not change appreciably. In addition, when we excluded the studies out of HWE, the statistical significance of the results did not change (Table 2).

## DISCUSSION

This is the 1st quantitative evaluation on *IFN-γ* +874T/A polymorphism and leukemia risk. Results based on the 420 leukemia cases and 767 matched controls from 8 studies suggested that *IFN-γ* +874T/A polymorphism were related to CML and CLL susceptibility. In this study, although associations between *IFN-γ* +874T/A polymorphism and overall leukemia risks were lacking, higher CLL susceptibility was detected in allelic model, codominant model, and dominant model, and results suggested T carries (patients with TT or TA genotype) have a decreased risk of CLL compared with AA genotype. On the contrary, results from dominant model indicated T carries have nearly 1.783-fold increased CML risk. To our best knowledge, this is the 1st evidence suggested *IFN-γ* +874T/A T allele increased cancer risk from meta-analysis.

T allele in the +874 position of *IFN-γ* is a protect genotype for CLL is biologically plausible. First, the +874 position, which is in strong linkage disequilibrium with the 12 CA repeat microsatellite in the noncoding region of the 1st intron of IFN-γ gene, controls the production of IFN-γ.^20^ As we known now, IFN-γ is a proinflammatory cytokine playing a pivotal role in both innate and adaptive immune responses, and has antitumor and antiproliferative effects.^6^ Therefore, it is understandable that T allele, which with high level IFN-γ production, may be a protect gene for CLL. Second, the DNA sequence containing the +874 T allele is the specific binding site for NF-κB.^21^ NF-κB knockout mice have been shown to have low values of IFN-γ and an increased susceptibility to infection.^22^ This polymorphism leading to the dysfunction of NF-κB pathway may cause oxidative damage, which also can increase the risk of cancer.^21^ Indeed, our finding is consistent with previous meta-analysis that T allele is a protect genotype among patients in breast cancer, cervical cancer, asthma, and leprosy.^23–26^

However, this study observed T allele is a risk genotype for CML. These results suggested 2 faces of IFN-γ in leukemia. Indeed, T allele is a risk genotype for diseases had been observed in bipolar disorder,^27^ and this is also not a new concept for the roles of cytokines in cancer. Transforming growth factor (TGF)-β has an ability to induce malignant behavior of normal fibroblasts, at the same time, it has profound growth-suppressive effects on many cells.^28^ Tumor necrosis factor (TNF), an extraordinarily pleiotropic cytokine, also displays pro- and antitumoral effects.^29^ A potential mechanism is localized inflammation may lead to an immunosuppressive and tolerogenic tumor microenvironment.^30^ For the dark side of IFN-γ, Zaidi and Merlino^31^ believed it may lie in the homeostatic functions of IFN-γ. Although high level of IFN-γ can protect normal cells from the collateral damage, these same mechanisms may also allow cancer cells to evade attack from the immune system.^31^ NK cells play a central role in tissue repair, however, Taniguchi et al^32^ found IFN-γ can enhanced resistance to NK cells, which was confirmed by another study and possible mechanism is higher metastatic ability of IFN-γ clones attributed to increased resistance to NK cells.^33^ But evidence so far for the roles of IFN-γ that may be more protumorigenic has been indirect, the exact mechanisms might IFN-γ behave as a “bad guy” in cancer need to be confirmed by further studies.

Genetic susceptibility is believed to be an important factor in the development of cancers.^34^ Meanwhile, the predisposition of every cancer types could be differentially influenced by genetic factors. Previous studies have showed the association between *IFN-γ* +874T/A polymorphism and cervical cancer,^35,36^ bladder cancer,^37^ liver cancer,^38^ and breast cancer,^39^ and suggested that T allele might be a protective genotype in cancer for TT genotype produces a high level of IFN-γ. Yet a recent meta-analysis showed no association between *IFN-γ* +874T/A polymorphism and cancer susceptibility,^40^ at the same time, the other meta-analysis found positive association in cervical cancer and breast cancer,^23,26^ which suggested that *IFN-γ* +874T/A polymorphism play different roles in different cancer types; therefore, the conclusion from a pooled analysis for association between *IFN-γ* +874T/A polymorphism and all cancer types may be unreliable. This meta-analysis confirmed this point. We found no significant association between *IFN-γ* +874A/T polymorphism and leukemia risk when all leukemia types were pooled into the meta-analysis, however, when stratified by leukemia types, results suggested T allele contributed completely opposite influence between CML and CLL. These results not only could partly explain the inconsistent results in different leukemia types but are also the reason why we conducted this study.

This study performed a subgroup analysis stratified by ethnicities and the significant associations were only found in the dominant model among Asians. Therefore, the genetic background may be another important factor contributed to the inconsistent results. Indeed, genetic background is distinct among different populations and our previous study had shown the genotype and allele frequencies of *IFN-γ* +874T/A were quite different among different regions and ethnicities.^4^ However, we noticed that there are 2 studies on CLL and 3 on CML in Caucasians, which may lead to negative results happened in the pooled analysis considering the dual role of *IFN-γ* +874T/A in CLL and CML.

Genome-wide association studies (GWAS) have successfully identified 13 loci associated with risk of CLL/small lymphocytic lymphoma before 2011.^41–43^ Then, Berndt et al^44^ discovered 10 independent SNPs in 9 novel loci in 2013 and Speedy et al^45^ identified five SNPs in new susceptibility loci in 2014 by GWAS separately. These studies suggest that additional risk loci should be identified by more studies. Our results suggested that rs2430561 in *IFN*-γ on chromosome 12q24 contributes to susceptibility to CML and CLL based on traditional genotyping methods, and these results have motivated us to carry out a GWAS focus on the SNPs on chromosome 12 to discover novel susceptibility loci for leukemia in the future.

In clinical practice, patients with leukemia are much less than that of lung cancer, liver cancer, breast cancer, and cervical cancer; therefore, the most limitation of this meta-analysis is that the present studies on susceptibility of leukemia are all with relative small sample sizes. As an example, we noticed the TA genotype frequency is only 1 in the study by Amirzargar et al,^10^ which can easily lead to false positive or negative results. Thus, we need to be taken into consideration when interpreting the findings. Further study with larger sample numbers will be helpful to improve the power for detecting the positive effects. In addition, other confounders, such as age and gender, were not adjusted because it is hard to get original data from author. In spite of these, our meta-analysis had some advantages. In the first place, this study greatly increased the statistical power compared with individual studies. There is one more point, no significant heterogeneity was detected in subgroup analysis stratified by leukemia types, together with publication bias and sensitivity analysis results indicated that our results were moderately robust.

## CONCLUSIONS

Taken together, results from this meta-analysis demonstrate that *IFN-γ* +874T/A polymorphism contribute to CML and CLL risk. However, our results are still needed to strengthen by further studies with larger sample sizes.

## Supplementary Material

Supplemental Digital Content
