# Supplementary material for: Association of Interferon Gamma +874T/A Polymorphism and Leukemia Risk: A Meta-Analysis
Source: Medicine (Baltimore). 2016 Mar 25;95(12):e3129. doi: 10.1097/MD.0000000000003129 (PMC4998384; doi:10.1097/MD.0000000000003129)
Supplement: Supplemental Digital Content [file medi-95-e3129-s001.doc]

| **Section/topic** | **#** | | **Checklist item** | | | **Reported on page #** | |
| --- | --- | --- | --- | --- | --- | --- | --- |
| **TITLE** | | | | | |  | |
| Title |  | | Identify the report as a systematic review, meta-analysis, or both. | | | Title, Page 1 | |
| **ABSTRACT** | | | | | |  | |
| Structured summary |  | | Provide a structured summary including, as applicable: background; objectives; data sources; study eligibility criteria, participants, and interventions; study appraisal and synthesis methods; results; limitations; conclusions and implications of key findings; systematic review registration number. | | | Abstract  Page 2 | |
| **INTRODUCTION** | | | | | |  | |
| Rationale |  | | Describe the rationale for the review in the context of what is already known. | | | Introduction  Paragraph 1,2,3,Page 3,4 | |
| Objectives |  | | Provide an explicit statement of questions being addressed with reference to participants, interventions, comparisons, outcomes, and study design (PICOS). | | | Introduction  Paragraph 4, Page 4 | |
| **METHODS** | | | | | |  | |
| Protocol and registration |  | | Indicate if a review protocol exists, if and where it can be accessed (e.g., Web address), and, if available, provide registration information including registration number. | | | Materials and Methods  Paragraph 1,2 .Page 3 | |
| Eligibility criteria |  | | Specify study characteristics (e.g., PICOS, length of follow-up) and report characteristics (e.g., years considered, language, publication status) used as criteria for eligibility, giving rationale. | | | Materials and Methods  Paragraph 2,3 .Page 4 | |
| Information sources |  | | Describe all information sources (e.g., databases with dates of coverage, contact with study authors to identify additional studies) in the search and date last searched. | | | Materials and Methods  Paragraph 4, Page 5 | |
| Search |  | | Present full electronic search strategy for at least one database, including any limits used, such that it could be repeated. | | | Materials and Methods  Paragraph 2, Page 3 | |
| Study selection |  | | State the process for selecting studies (i.e., screening, eligibility, included in systematic review, and, if applicable, included in the meta-analysis). | | | Materials and Methods  Paragraph 3, Page 3 | |
| Data collection process |  | | Describe method of data extraction from reports (e.g., piloted forms, independently, in duplicate) and any processes for obtaining and confirming data from investigators. | | | Materials and Methods  Paragraph 3, Page 3 | |
| **ection/topic** | | **#** | | **Checklist item** | **Reported on page #** | | |
| Data items |  | | List and define all variables for which data were sought (e.g., PICOS, funding sources) and any assumptions and simplifications made. | | | | Materials and Methods, Page 5 |
| Risk of bias in individual studies |  | | Describe methods used for assessing risk of bias of individual studies (including specification of whether this was done at the study or outcome level), and how this information is to be used in any data synthesis. | | | | Materials and Methods, Page 5 |
| Summary measures |  | | State the principal summary measures (e.g., risk ratio, difference in means). | | | | Materials and Methods, Page 5 |
| Synthesis of results |  | | Describe the methods of handling data and combining results of studies, if done, including measures of consistency (e.g., I^2^) for each meta-analysis. | | | | Materials and Methods, Page 5 |
| Risk of bias across studies | |  | | Specify any assessment of risk of bias that may affect the cumulative evidence (e.g., publication bias, selective reporting within studies). | | | Materials and Methods, Page 5 |
| Additional analyses | |  | | Describe methods of additional analyses (e.g., sensitivity or subgroup analyses, meta-regression), if done, indicating which were pre-specified. | | | Materials and Methods, Page 5 |
| **RESULTS** | | | | | | |  |
| Study selection | |  | | Give numbers of studies screened, assessed for eligibility, and included in the review, with reasons for exclusions at each stage, ideally with a flow diagram. | | | Results, page 6 |
| Study characteristics | |  | | For each study, present characteristics for which data were extracted (e.g., study size, PICOS, follow-up period) and provide the citations. | | | Results  page 6 |
| Risk of bias within studies | |  | | Present data on risk of bias of each study and, if available, any outcome level assessment (see item 12). | | | Results  page 7 |
| Results of individual studies | |  | | For all outcomes considered (benefits or harms), present, for each study: (a) simple summary data for each intervention group (b) effect estimates and confidence intervals, ideally with a forest plot. | | | Results  Table 2,Figure 2 |
| Synthesis of results | |  | | Present results of each meta-analysis done, including confidence intervals and measures of consistency. | | | Results  Table2 |
| Risk of bias across studies | |  | | Present results of any assessment of risk of bias across studies (see Item 15). | | | Results  page 7 |
| Additional analysis | |  | | Give results of additional analyses, if done (e.g., sensitivity or subgroup analyses, meta-regression [see Item 16]). | | | Results  page 7 |
|  | |  | |  | | |  |
| **ection/topic** | | **#** | | **Checklist item** | **Reported on page #** | | |
| **DISCUSSION** | | | | | | |  |
| Summary of evidence | |  | | Summarize the main findings including the strength of evidence for each main outcome; consider their relevance to key groups (e.g., healthcare providers, users, and policy makers). | | | Discussion  page 7,8 |
| Limitations | |  | | Discuss limitations at study and outcome level (e.g., risk of bias), and at review-level (e.g., incomplete retrieval of identified research, reporting bias). | | | Discussion  page 9,10 |
| Conclusions | |  | | Provide a general interpretation of the results in the context of other evidence, and implications for future research. | | | Discussion  page 10 |
| **FUNDING** | | | | | | |  |
| Funding | |  | | Describe sources of funding for the systematic review and other support (e.g., supply of data); role of funders for the systematic review. | | | page 10 |

*From:*  Moher D, Liberati A, Tetzlaff J, Altman DG, The PRISMA Group (2009). Preferred Reporting Items for Systematic Reviews and Meta-Analyses: The PRISMA Statement. PLoS Med 6(6): e1000097. doi:10.1371/journal.pmed1000097

For more information, visit: **www.prisma-statement.org**.

**S2 file: List of full-text excluded article**

In the literature search process, 13 full-text articles were assessed for eligibility and 6 irrelevant papers were excluded as following reasons:

**1 for IFN-alpha polymorphism[1]**

**1 for FAS promoter polymorphism [2]**

**1 for IFN-γ type 2 receptor polymorphism [3]**

**1 for IFN-γ 13-CA-repeat polymorphism [4]**

**1 for IFN-γ rs2069727 polymorphism [5]**

**1 for human T-lymphotropic virus type 1 infection [6]**

1. Basturk B, Evke E, Karakus S, Tunali A: Potential risk factor of interleukin-10 gene polymorphism in acute myelogenous leukemia. *UHOD - Uluslararasi Hematoloji-Onkoloji Dergisi* 2005, 15(2):57-62.

2. Farre L, Bittencourt AL, Silva-Santos G, Almeida A, Silva AC, Decanine D, Soares GM, Alcantara LC, Jr., Van Dooren S, Galvao-Castro B *et al*: Fas 670 promoter polymorphism is associated to susceptibility, clinical presentation, and survival in adult T cell leukemia. *Journal of leukocyte biology* 2008, 83(1):220-222.

3. Hattori H, Matsuzaki A, Suminoe A, Ihara K, Nagatoshi Y, Sakata N, Kawa K, Okamura J, Hara T: Polymorphisms of transforming growth factor-(beta)1 and transforming growth factor-(beta)1 type II receptor genes are associated with acute graft-versus-host disease in children with HLA-matched sibling bone marrow transplantation. *Bone marrow transplantation* 2002, 30(10):665-671.

4. Jaskula E, Dlubek D, Duda D, Bogunia-Kubik K, Mlynarczewska A, Lange A: Interferon Gamma 13-CA-Repeat Homozygous Genotype and a Low Proportion of CD4+ Lymphocytes Are Independent Risk Factors for Cytomegalovirus Reactivation with a High Number of Copies in Hematopoietic Stem Cell Transplantation Recipients. *Biology of Blood and Marrow Transplantation* 2009, 15(10):1296-1305.

5. Morrison BA, Ucisik-Akkaya E, Flores H, Alaez C, Gorodezky C, Dorak MT: Multiple sclerosis risk markers in HLA-DRA, HLA-C, and IFNG genes are associated with sex-specific childhood leukemia risk. *Autoimmunity* 2010, 43(8):690-697.

6. Rocha-Junior MC, Haddad R, Ciliao Alves DC, de Deus Wagatsuma VM, Mendes-Junior CT, Deghaide NHS, Takayanagui OM, Covas DT, Donadi EA, Kashima S: Interleukin-18 and interferon-gamma polymorphisms are implicated on proviral load and susceptibility to human T-lymphotropic virus type 1 infection. *Tissue antigens* 2012, 80(2):143-150.
